# An overview of the role of neutrophils in innate immunity, inflammation and host-biomaterial integration

**DOI:** 10.1093/rb/rbw041

**Published:** 2017-02

**Authors:** Gretchen S. Selders, Allison E. Fetz, Marko Z. Radic, Gary L. Bowlin

**Affiliations:** 1 Department of Biomedical Engineering, University of Memphis, Memphis, TN, USA; 2 Department of Microbiology, Immunology and Biochemistry, University of Tennessee Health Science Center (UTHSC), Memphis, TN, USA, 858 Madison Ave, Room 201 Molecular Science Building, Memphis, TN 38163, USA

**Keywords:** neutrophil, tissue engineering, NETosis, tissue regeneration, host response, inflammation

## Abstract

Despite considerable recent progress in defining neutrophil functions and behaviors in tissue repair, much remains to be determined with regards to its overall role in the tissue integration of biomaterials. This article provides an overview of the neutrophil’s numerous, important roles in both inflammation and resolution, and subsequently, their role in biomaterial integration. Neutrophils function in three primary capacities: generation of oxidative bursts, release of granules and formation of neutrophil extracellular traps (NETs); these combined functions enable neutrophil involvement in inflammation, macrophage recruitment, M2 macrophage differentiation, resolution of inflammation, angiogenesis, tumor formation and immune system activation. Neutrophils exhibit great flexibility to adjust to the prevalent microenvironmental conditions in the tissue; thus, the biomaterial composition and fabrication will potentially influence neutrophil behavior following confrontation. This review serves to highlight the neutrophil’s plasticity, reiterating that neutrophils are not just simple suicidal killers, but the true maestros of resolution and regeneration.

## Introduction

The most important aspect of biomaterial design is the tailoring of the device to meet the needs of the body’s innate immune system and wound healing response. Most certainly in tissue engineering, the biomaterial is designed to biomimic a particular tissue structure, and by mimicking that tissue structure, accomplish appropriate tissue function and ultimately, tissue repair and biomaterial integration. However, the intended application is heavily affected by the body’s response to the material composition, structure, surface properties and the inflammatory response initiated by either the prior tissue damage or the implantation of a device. No matter the material or design, there is invariably a host response. Upon implantation, the innate immune system initiates a series of events, including blood plasma protein deposition and cell recruitment/attachment (namely leukocytes), which ultimately influence the success of the biomaterial [1, 2]. Tailoring the biomaterial to induce or align the cells in the affected tissue along a regenerative pathway, while simultaneously degrading parallel to the tissue integration, is the greatest advance that can be made in the field of biomaterial design, and ultimately, for tissue engineering as a whole. In order to fully realize the biological response to the biomaterial, it is critical to understand not only the body’s innate wound healing response, but also the synergy of the cell types involved, beginning with the underappreciated neutrophil. Despite considerable recent progress in defining neutrophil functions and behaviors in tissue repair, much remains to be determined with regards to their overall role in tissue integration of biomaterials.

This article serves as an introduction to the current understanding of neutrophils, a review of recent research and literature, and an evaluation of the remaining unknowns. It discusses the immediate need to better tailor the currently lacking biomaterials to stimulate neutrophils to affect a host-appropriate response and a regenerative outcome. *‘In vivo* veritas’ means ‘within the living, there lies the truth’, implying that the only clear and reasonable approach for overcoming the current limitations of biomaterial design and tissue engineering is to realize the host’s innate response and aim to biomimic the naturally occurring series of events to promote tissue integration and regeneration. As neutrophils are primarily the first cells to confront the biomaterial, research must begin with the initial phase of inflammation to better understand how this primary interaction sets the stage for a cascade of events.

## The wound healing response

Response to an injury following the implantation of a biomaterial is largely based on the extent and size of the injury or implant, anatomical [tissue] location of the implant, loss of basement structures, blood-biomaterial interactions, provisional matrix production and the severity of the inflammatory response [3, 4]. Acute inflammation is a normal and necessary function of our innate immune system. It is initiated by pathogen presence or tissue damage (i.e. biomaterial implantation) and is the immune system’s first line of defense in evading infection and attacking a foreign agent, beginning with the neutrophil. Biomaterials are foreign objects and, by definition, elicit an immune response, but the design (composition, fabrication, size and topography) influences the interacting cell(s) behavior and recruitment, determining whether or not the particular biomaterial evokes an acute, short-lived, normal and necessary phase of inflammation leading to tissue regeneration or a sustained immune system response (chronic inflammation) leading to accelerated material degradation and tissue destruction.

Biocompatibility is a critical aspect of biomaterial design and is considered inversely related to the magnitude and duration of the homeostatic mechanisms that control the host response [3]. Poor biocompatibility often results in fibrous encapsulation. It is considered by many a failure of the device if it becomes fibrotically encapsulated, regardless of functional capabilities. This inflammatory response is modulated partly by the neutrophil as there is an acute confrontation of neutrophils and the biomaterial through blood-material interactions resulting from injury to the surrounding vasculature. Injury stimulates vasodilation, and there is an increase in vascular permeability, aiding in neutrophil delivery to the site [3]. Subsequently, clots are formed through the coagulation cascade, and the resulting adsorption of proteins on the biomaterial surface is commonly considered to be provisional matrix deposition [3]. The provisional matrix includes chemoattractants which can stimulate or recruit other cells (i.e. neutrophils) that modulate macrophage recruitment [4]. This orchestrated response to an implanted biomaterial also includes the coagulation cascade events, complement system, fibrinolytic system, kinin-generating system, platelets and many other components that together play a crucial role in stemming blood loss and delivering neutrophils to the site of injury [3]. More importantly, the platelets and neutrophils will primarily be the first cells of the innate immune system to interact with the implanted biomaterial; platelets function in a variety of manners (formation of platelet plug, bind via cell-surface receptors, and secrete cytokines and antimicrobial peptides) and their presence alongside the neutrophil in the initial phase of inflammation, indicates that these two cell types play a critical role in the onset of inflammation [2].

Historically, literature claims that neutrophils predominate during the first hours of the inflammatory response and are short-lived with minimal impact compared to succeeding cell types, namely monocytes and macrophages [3]. However, the number of monocytes/macrophages in a wound directly correlates to the number of neutrophils present, and this suggests that the neutrophils are orchestrating the recruitment of resolving cells. Although originally thought to survive 24 h or less (7–12-h half-life) upon migration into tissue, neutrophils remain present in the wound site for extended periods of time, up to 3 days, composing the ‘most important feature’ of the acute inflammatory response that clean up debris via phagocytosis [3, 5, 6]. In fact, inflammation often counteracts the apoptosis of neutrophils, eliciting a prolonged neutrophil recruitment and presence [7]. The longevity of neutrophil presence in the wound site is supported by evidence of both continuous recruitment of neutrophils to the site as well as inhibition of the normal spontaneous apoptosis of neutrophils during the resolution of inflammation, prolonging and increasing severity of the inflammatory response [7, 8]. In an appropriate (or potentially acute) inflammatory response, the neutrophil is needed for performing necessary resolution steps such as phagocytosis and cell recruitment, but its timely removal is also critical to the maintenance of an acute response. The three step phagocytic process of recognition and attachment, engulfment and subsequent degradation of a foreign agent performed via the neutrophil can be an unsuccessful attempt to clean up biomaterials, mostly due to their size [3]. As a result, neutrophil interaction compounded by the presence of frustrated macrophages (macrophages which produce reactive oxygen species (ROS) and degradative enzymes) can lead to chronic inflammation, fibrosis, and implant rejection, which is undesirable for tissue repair and integration [4].

Chronic inflammation is hallmarked by the prolonged presence of foreign body giant cells, or the fusion of frustrated macrophages, as wound healing progresses [1, 4]. The fusion of frustrated macrophages to form foreign body giant cells is the immune system’s struggle to attack and degrade large implants. Originating in the wound site by the fusion of many frustrated macrophages, these cells can remain attached to the biomaterial surface for extended periods of time, creating an impermeable layer between host and material and eventually leading to destruction and/or failure of the device [3, 9]. If the wound site has become a chaotic arena for foreign body giant cells, fibrosis, the last stage of the healing response, is dominated by ‘tissue replacement’ with connective fibrous-like tissues. However, if the response has been more regulated, the last stage of healing consists of ‘tissue regeneration’ or restoration of lost tissue by parenchymal cells. Most notably, this regeneration of lost tissue can be connected to the orchestration of certain pathways beginning with the neutrophil, whose importance in the maintenance and restoration of homeostasis is indicated and further highlighted by numerous diseases (chronic inflammatory and autoimmune) associated with its deficiency and excess [3, 10, 11]. Diseases such as Lupus and chronic periodontal disease are impacted by altered neutrophil functionality (low density of granules, diminished phagocytic capabilities and enhanced NET formation) rather than deficiency and excess [12, 13]. It has also been seen that the production rate of neutrophils from bone marrow can see a 10-fold increase during an infection state, which demonstrates the neutrophil’s affinity for and response to inflammatory signals [14]. It is possible that biomaterials can induce these aspects of chronic inflammation and prolonged neutrophil presence and frustrated phagocytosis. Therefore, the key is to optimize the biomaterial design by understanding the neutrophil-macrophage relationship and induce synergy between them in order to create regenerative outcomes, rather than frustrated phagocytosis, formation of foreign body giant cells and fibrous tissue encapsulation.

## Neutrophils in inflammation

Neutrophils are polymorphonuclear lymphocytes produced daily by the body in large quantities (10^11^ produced by the bone marrow each day) and reside mainly in the peripheral vasculature [15]. The multi-lobe nucleus aids neutrophils in movement through tight gaps formed between other cells or within narrow pores in the extracellular matrix (ECM); the option for the cell to align portions of its nucleus in a linear fashion facilitates neutrophil migration from the blood into tissue better than cells with larger spherical nuclear regions [16, 17]. Neutrophils have been viewed as swift, short-lived effector cells of the immune system, which serve solely to perform phagocytosis, recruit other effector cells and commit cell suicide via apoptosis [2, 6]. Moreover, neutrophils have been considered the major innate immune cells responsible for tissue damage and harm to the host tissues; however, this is far from a complete picture, as neutrophils have even more flexibility to adjust to the prevalent microenvironmental conditions in a distressed tissue [18, 19].

Although classically considered to be ‘only’ effector cells, neutrophils interact with other cells, influencing, recruiting, and secreting signals for surrounding immune and humoral cells [20]. Neutrophils function in three primary capacities: generation of oxidative bursts, release of granules, and formation of NETs [19, 21]. First and foremost, they are highly capable phagocytes and have been known to release lytic enzymes and ROS to cleave bacterial virulence factors as immune effector cells of regeneration [15, 20]. Neutrophil release of ROS and antimicrobial agents kill the pathogens either surrounding them or via release of such agents within NETs [20–22]. The granules released contain a variety of components aiding in both pro- and anti-inflammatory behaviors, killing pathogens via proteases and providing substances such as matrix metalloproteinases (MMPs) to allow for matrix reprogramming, angiogenesis and regeneration [11, 23, 24]. NET formation facilitates enhanced pathogen trapping and destruction as well as release of granule contents [6, 25, 26]. Due to recent research and discovery of neutrophil function, it is now known that neutrophils are capable of much more than playing the role of suicidal killers, highlighting them as the ‘key’ effector cells of the innate immune system, the maestros of resolution and regeneration.

Neutrophils produce many anti-inflammatory factors including chemokines and cytokines, often through the release of their four different cytosolic granules [20]. The factors contained within the primary (azurophilic), secondary (specific), tertiary (gelatinase), and secretory vesicles (Table 1) function in a variety of ways and may play many powerful roles in immune system guided *in situ* regeneration (20). The primary granules release certain factors such as myeloperoxidase (MPO) and neutrophil elastase (NE). Secondary granules release others including peptidoglycan recognition protein (PRGP), M-ficolin and lactoferrin. Tertiary granules release matrix degrading proteins such as MMP-9. Of particular note, MMP-9 is uniquely released by neutrophils tissue inhibitor of metalloprotease-free (TIMP-free) (TIMP-free MMP-9) [43]. MPO and NE are responsible for antimicrobial activity. M-Ficolin and PRGP (from both secondary and tertiary granules) are responsible for specific bacterial and bactericidal activity [6]. NE, with MPO, and MMP-9 are the main tissue destructive agents heavily involved in matrix degradation. In excessive amounts, and especially in neutrophil presence, these agents have been implicated in unwanted accelerated biomaterial degradation [9, 44, 45]. MMP production is upregulated in response to pathogen presence, tissue necrosis factor (TNF) alpha (TNF-α), and other inflammatory mediators [44]. Furthermore, MMPs functioning as gelatinases and collagenases, can break down connective tissues alone as well as work in tandem with other proteases to attack the microenvironment. Conversely, in the appropriate amounts, MMPs can be associated with matrix reprogramming, angiogenesis and tissue remodeling, consistent with resolution of the inflammatory response [6]. Last, secretory vesicles have been reported to be released upon neutrophil engagement with endothelial cells [46].

**Table 1.** Neutrophil granules and their factors of interest. Chemokine production and secretion play a significant role in cellular migration, wound healing, hematopoiesis, angiogenesis, and tumor metastasis, all critical to the neutrophil’s function [4]

Granule	Factor of Interest	Function	Role in Immune System Guided *In Situ* Regeneration
Primary *Azurophilic granules*	NE *(serine protease)*	Degrades collagen-IV and elastin within ECM [15]	Positive feedback loop for the inflammatory response [27]
		Targets bacteria’s virulent vectors [28]	
		Up-regulates expression of TLR 4 expression in monocytes [27]	
		Tissue remodeling [4]	
	Defensins	Disrupts cytoplasmic membrane of microbes and induces migration of naïve T cells and immature DCs [29]	Active adaptive immunity to combat infection [D. 29]
		Induces chemotaxis of CD4+ and CD8+ cells (30)	Links innate and adaptive immunity through the neutrophil (30)
	MPO *(peroxidase)*	Production of antimicrobial oxidants [31]	Facilitates NET release [32]
		Enables translocation of NE to the nucleus [32]	
		Reacts with H_2_O_2_ which increases toxic potential by inducing the formation of hypochlorous acid (chlorination products, tyrosine radicals and reactive nitrogen intermediates) [15, 30]	
	Lysozyme	Cleaves peptidoglycan polymers of bacterial cell walls [30]	
	Bactericidal/permeability increasing protein (BPI)	Kills gram-negative bacteria at non-molar concentrations by binding to negatively charged residues of LPS which promotes bacterial attachment and allows for phagocytosis [15, 30]	
		Endotoxin-neutralizing proteins [15]	
	Proteinase 3	Induces activation of epithelial cells, endothelial cells, macrophages, lymphocytes, and platelets [30]	
	Cathepsin G *(serine protease)*	Kills pathogens [15]	Tissue remodeling [15]
		Degrades ECM proteins [15]	
		Induces activation of epithelial cells, endothelial cells, macrophages, lymphocytes, and platelets [30]	
	Azurocidin	Induces chemotaxis of CD4+ and CD8+ cells [30]	
		Antimicrobial activity [15]	
	Vitronectin	Promotes neutrophil adhesion and migration through interaction with integrins [33]	
		Inhibits apoptosis of neutrophils [33]	
Secondary* Specific granules*	Lactoferrin	Wide range of microbicidal activity against pathogens (15)	
		N-terminal amphipathic α-helical region [30]	
		Iron-binding proteins and impairs bacterial growth (gram − and +) by sequestration of iron [30]	
	Collagenase (MMP-1 and MMP-8)	Degrades major structural components of ECM [30]	MMP-8 has been deemed a tumor-protective protein, possibly to be an anti-tumor agent against MMP-9 [34]
		Responsible for loss of vascular basement membranes during neutrophil extravasation and migration [30]	
	M-Ficolin	Interacts with microbial entities and activates the lectin pathway of the complement cascade [4]	
	Neutrophil gelatinase associated lipocalin	Antibacterial activity through sequestration of ferric-siderophore complexes [30]	Is produced commonly by neutrophils in normal, inflamed, and neotissue [30]
		Strongest iron chelators known [35]	Plays a role in iron-depleting strategy affecting bacterial growth [30]
	Human cathelicidin antimicrobial protein-18 (hCAP-18)	Antimicrobial peptide (−/+), induces chemotaxis of neutrophils, T cells and monocytes when isolated from cathelin propiece [30]	During wound healing, insulin-like growth factor 1 (IGF-1) induces secretion of hCAP-18 in keratinocytes and hCAP-18 is constitutively expressed in monocytes and lymphocytes elsewhere [30]
	Flavocytochrome b_558_	Terminal electron carrier of the assembled respiratory burst oxidase [30]	
	Lysozyme	Binds LPS and reduces cytokine production [30]	
		Bactericidal activity against non-pathogenic bacteria [30]	
	Secretory leukocyte protease inhibitor (SLPI)	Neutralizes elastase and cathepsin G., activates MMPs, inhibits macrophage MMPs and tumorigenesis; absence of SLPI associated with reduced ECM production and poor healing [36]	
	Pentraxin 3	Antimicrobial properties [37]	Stimulated by LPS, neutrophil activation etc. and can continue to be released in response to inflammatory cytokines [37]
		Microbial recognition [4]	
	NADPH oxidase	Aids respiratory burst upon neutrophil activation and subsequent ROS production/release [15]	
	Leukolysin (MMP-25) (10% of total leukolysin present in cell)	Degrades major structural components of ECM [30]	
		Loss of vascular basement membranes during neutrophil extravasation and migration [30]	
Tertiary *Gelatinase granules*	Gelatinases A and B (MMP- 2 and MMP-9)	Degrades major structural components of ECM [30]	Inhibition of gelatinases results in suppressed neutrophil attachment and migration [38]
		Loss of vascular basement membranes during neutrophil extravasation and migration [30]	Excessive amounts of MMP-9, potent stimulator of angiogenesis, seen in N2 neutrophils plays a role in invasive tumor growth [34]
		Tissue remodeling [4]	
	Flavocytochrome b_558_	Terminal electron carrier of the assembled respiratory burst oxidase [30]	
	Arginase-1	Inhibits T cell proliferation [39]	Lack of Arginase-1 is associated with reduced healing, inflammation, increased collagen deposition and mast cell migration [40]
	Leukolysin (MMP-25) (40% of total leukolysin present in cell)	Degrades major structural components of ECM [30]	Allows for neutrophil migration and matrix reprogramming
		Loss of vascular basement membranes during neutrophil extravasation and migration [30]	
Secretory Vesicles	β_2_-integrin CD11b/CD18 (Mac-1, CR3)	Promotes apoptosis of neutrophils [41]	When mobilized, there is a shedding of L-selectin from neutrophil’s surface which allows for neutrophil firm contact with the vascular endothelium *in vivo* [30]
			Increased apoptosis of neutrophils can lead to resolution of inflammation [41]
	Formylated bacterial peptides (fMLP-receptors)	G-PCR	In LPS stimulated neutrophils, fMLP can inhibit TNF-α providing an anti-inflammatory effect on monocytes and macrophages [42]
		Pro-inflammatory agent [42]	

Recent literature reports that neutrophils survive much longer upon migrating into tissue, 3 days and possibly longer, due to enhanced survival and continuous recruitment. Previous literature claims they are present for the first 7–24 h post-injury (expected with surgical trauma) [6, 47]. However, this timeline has been challenged by evidence of continuous neutrophil recruitment, and in an *in vivo* study where Jhunjhunwala *et al.* [47] observed a ‘30–500-fold increased neutrophil presence’ after 2 week response to a peritoneal implant in mice .

### Neutrophil migration

The migration of neutrophils to the site of injury has been portrayed as a multiphase process, beginning with initial neutrophil migration and ending with reverse migration, or return to the vasculature [48]. The neutrophil presence is also amplified and sustained via neutrophil recruitment, either by the active neutrophils themselves or the surrounding tissue-resident macrophages. Furthermore, differentiation of migrated monocytes into macrophages is guided by cytokines, chemokines (Table 1), and lipids released via the neutrophil’s granules [46]. This demonstrates the ‘polarizing effect’ of the neutrophil granular contents on functional macrophage phenotypes [46]. Even more recent literature provides evidence that neutrophils can become polarized themselves in response to certain signals, synonymous to macrophage polarization, with distinctive phenotypes (N1 and N2) resulting in very different effects on the immune system [2, 6].

Upon the occurrence of tissue damage (i.e. implantation of a biomaterial), neutrophils migrate out of circulation and begin crosstalk with immune and non-immune cells. Neutrophils can be activated or signaled to migrate in a variety of ways, including microbial presence, recognition of and activation via *N*-formyl peptides (such as formylmethionyl-leucyl-phenylalanine (fMLP)), Toll-like receptors (TLRs) and G-protein coupled receptors (G-PCR) [48]. fMLP peptides can either come from bacterial proteins or from tissue damage, both of which can activate neutrophils [48]. Additionally, they can be activated via formyl peptide receptor 1 which is directly related to bacteria, marking neutrophils as possessors of pattern recognition receptors (PRRs), like TLRs, which are capable of recognizing pathogens, a crucial attribute of a key effector cell of the immune system [6]. They express most TLRs and release many chemokines that recruit additional neutrophils and influence the function of neutrophils, like the production of ROS and lytic enzymes, which destroy pathogens and foreign agents, the main reason for the common labeling of neutrophils as destructive cells [27, 49]. The presence of granulocyte colony stimulating factor (G-CSF), TNF and Type I and II interferons (IFNs) can recruit and/or activate neutrophils [6].

Upon stimulation of neutrophils, there is a secretion of CXC-chemokines, which are responsible for chemotaxis of close-by neutrophils to the site. The migration of neutrophils to the site of injury has been described as a three phase process, including neutrophil forward migration, neutrophil recruitment amplification, and reverse migration [48]. The initial early recruitment of neutrophils is caused by damage-associated molecular patterns (DAMPs), which the neutrophil detects with its PRR [48]. DAMPS can include DNA, proteins, ECM components and *N*-formyl peptides, all of which can be the product of tissue damage or a bacterial agent. More distant neutrophils are attracted via CXCL-chemokines (CXCL8 family of chemokines) [48]. After initial migration, the neutrophil recruitment becomes amplified via leukotriene B4 (LTB4) and CXCL8 chemokines [48]. The third phase is characterized by the removal of neutrophils from the area either by phagocytosis via macrophages, apoptosis or reverse migration. Reverse migration is described as a process by which neutrophils return to the vasculature in a phenomenon deemed reverse transendothelial migration [48]. Although there has been evidence that neutrophils can migrate away from a chemoattractant, there is concern that neutrophils may relocate and create a new inflammation site [48].

### Neutrophils and cell recruitment

In order to promote the chemotaxis of other immune cells, neutrophils also secrete CC-chemokines responsible for monocyte recruitment and possibly resolving and repairing entities through monocyte differentiation into macrophages, secretion of pro-inflammatory cytokines such as IL-8 as well as anti-inflammatory cytokines such as IL-10. Neutrophils also release immunoregulatory cytokines such as IFN-γ, which recruits macrophages, and G-CSF, which ultimately stimulates neutrophil production and aids in extended neutrophil presence, and many other factors (reference Table 1 for more factors) [6]. Neutrophils either produce agents or recruit and effect other immune cells, which ultimately aid in the enhanced recruitment of neutrophils and their prolonged presence to allow for continued orchestration of the innate healing response.

**Figure 1.**
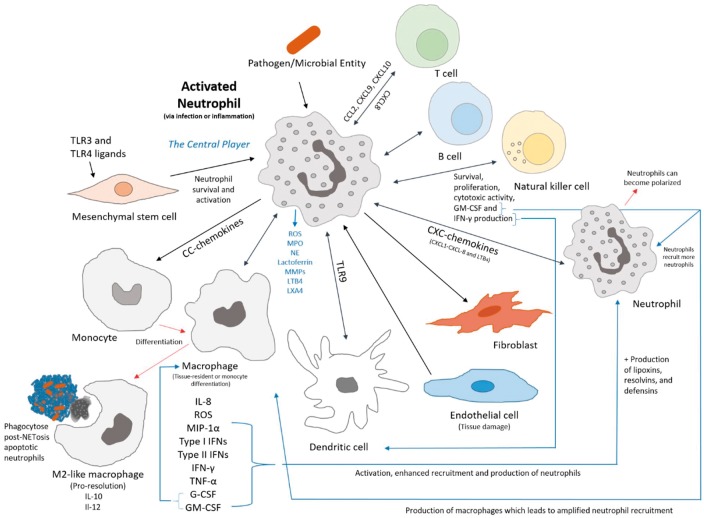
Neutrophil crosstalk with immune and humoral cells and relevant chemical signals that influence and compose the inflammatory response and pathway to resolution via the neutrophil.

Having assembled a list of the various key neutrophil-derived factors and cytokines (Table 1) produced in the wound microenvironment, this synopsis demonstrates that neutrophils are capable of much more than the previously defined, simple suicidal killers. This list also indicates that because of its ability to adopt either an immunoregulatory, pro-inflammatory (N1), or anti-inflammatory (N2) phenotype, as regulated by the microenvironment, and because of its role as first recruited cell at the injury site, the neutrophil must be the central player in modulating the innate immune system and host response. Furthermore, neutrophils are involved in the activation and recruitment of natural killer (NK) cells, dendritic cells (DCs) (via TLR9), and mesenchymal stem cells [6, 15, 18, 22]. The activation of the neutrophil can influence the recruitment, stimulation or function of macrophages, DCs, T cells, B cells, NK cells (Fig. 1), causing increased antimicrobial activity, increased activation and maturation of neutrophils, survival and proliferation of neutrophils and increased cytokine production [6, 18]. Additionally, chemokines, cytokines, and other signals produced by neutrophils and immune cells can impact neutrophil function, such as LTB4 increasing surface expression of NE on active neutrophils [50]. The understanding of the molecular mechanisms that control the neutrophil migration, response, phenotype and overall complex cell recruitment cycle is of utmost importance to understanding the host-biomaterial interface (Fig. 1). Neutrophil-based orchestration of the relevant cell types at the host-biomaterial interface can provide insight regarding a template’s regenerative potential.

## NET formation and the neutrophil’s unique form of cell death (NETosis)

**Figure 2.**
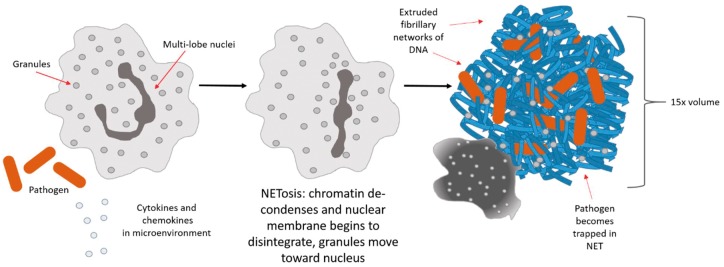
Schematic of the neutrophil undergoing NETosis.

In addition to the neutrophil’s ability to perform phagocytosis and recruit multiple cell types, neutrophils can release NETs via two pathways. Vital NET formation, although less common, occurs via mitochondrial DNA extrusion and the cell remains intact [4, 18, 51]. In contrast, NETosis and cell disintegration occurs via a morphologically different and novel form of cell death deemed NETosis, that is quite different from the classical form of cell death, apoptosis (Fig. 2) [52]. NETs are extruded as fibrillary networks composed of chromatin with entangled histones and coated with proteins from all types of neutrophil granules (primary, secondary and tertiary) that are anchored to the neutrophil’s body [22, 53]. NET formation can be elicited by the neutrophil’s exposure to cytokines, microbes or microbial products [21, 49]. The NETosis mechanism begins when an activated neutrophil flattens and attaches to a substrate. NE is released and in the process the nucleus loses its lobules and its chromatin decondenses while the granules mobilize towards the decondensing chromatin and disintegrate [15, 20–22, 54, 55]. The neutrophil then rounds back up and contracts until the cell membrane ruptures, and the NET is ejected [21, 22]. The resulting, web-like structure occupies nearly 15 times the volume (Fig. 2) of the original cell and appears cloud-like [22]. The voluminous, sticky NET formation serves to not only trap the pathogen, but also to localize the antimicrobial agents to prevent dilution, amplify their combined capabilities, and minimize damage to the surrounding tissue [25].

It has also been observed that deimination of histones in the neutrophil serve as a marker of neutrophil activation and a switch from a more tolerant (destined to return to the bone marrow and undergo apoptosis) to an autoimmune state (pro-inflammatory) [52]. When both neutrophil-like differentiated HL-60s and freshly isolated, human peripheral blood neutrophils were exposed to a variety of stimulants, powerful and swift deimination of histone H3 was seen, an event that is observed namely when the neutrophil evades apoptosis and instead, proceeds to a stage of NET extrusion [52]. It may also be an indication that histone entanglement within NETs enhances the neutrophil functional capacity [52].

**Figure 3.**
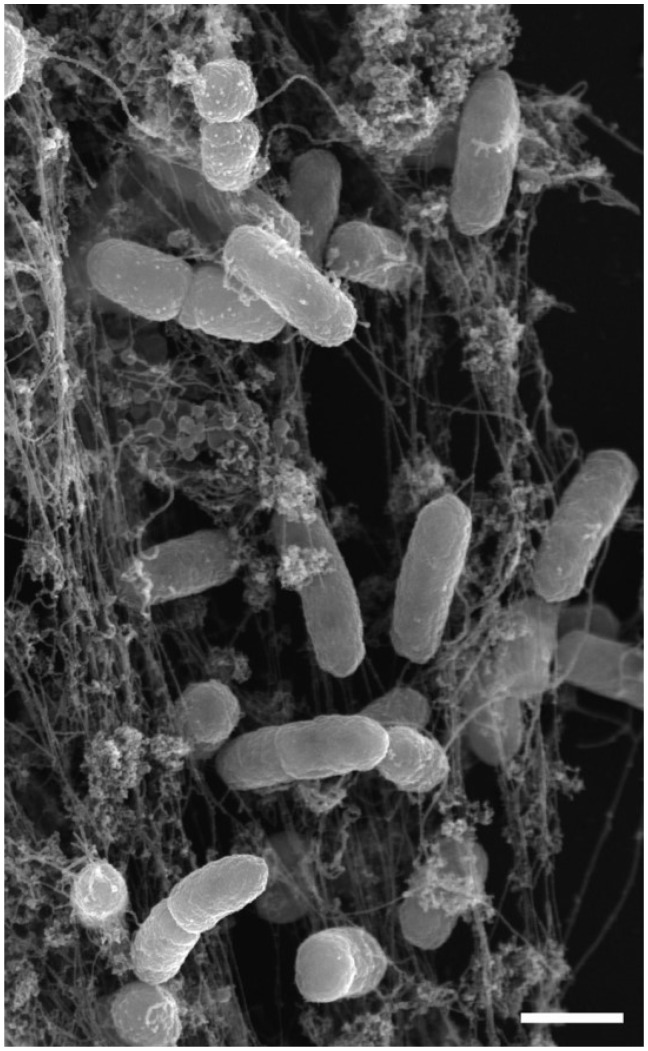
Scanning electron micrograph (scale bar represents 1 µm) of NETs trapping/entangling pathogens [22]. Reprinted with permission from original publisher. © 2012 Brinkmann and Zychlinksy. Journal of Cell Biology. 198:773-783,doi:10.1083/jcb.201203170.

NET formation results in a colossal, yet regulated release of histones which enhances antimicrobial activity, further extending the functionality of NETs [25]. One group has presented a possible connection of these extruded histones contained within the NETs and the activation of TLR2 and TLR4, suggesting yet another pathway for immune system activation along with neutrophil release of immunoregulatory factors, macrophage recruitment of neutrophils and bone marrow-derived mesenchymal stem cells’ anti-apoptotic actions activated via TLR3 and TLR4 [6, 22, 56]. However, NETs namely exist to trap pathogens (Fig. 3

) and force them to interact with the ROS and lytic enzymes with intent to destroy such pathogenic agents [22]. Furthermore, neutrophils release NETS when they are unable to phagocytose a noxious stimuli [54, 57]. This form of NET extrusion may be considered synonymous to the formation of foreign body giant cells by the fusion of frustrated macrophages.

NETs are capable of binding to both Gram-positive and Gram-negative bacteria and quickly degrade bacterial agents due to their granule contained proteases [18]. NET formation can be rapid, happening within minutes of activation, triggered by the presence of ROS, but is almost always the result of the cell undergoing a specialized form of cell death thus terming the behavior, NETosis, or cell death associated with NET extrusion [6, 18, 22, 25]. It has also been seen that following NETosis, anucleated neutrophils are able to continue phagocytosis and degranulation [15, 26]. Although protease dispersal via NETs is implicated in further tissue destruction, the role of proteases is not limited to cleaving bacterial factors, as they are potent antimicrobial agents that can actually end up neutralizing their own effects [25, 58]. NET formation can also be induced by platelets in the vascular periphery upon vessel wall damage; this serves to aid platelets and endothelial cells with trapping pathogens from the vasculature, preventing systemic infection. However, this can negatively impact healthy tissue by contributing to vascular inflammation and possible thrombosis as triggered by tissue factor contained within extruded NETs [15, 59]. Therefore, NETs can function to physically trap bacteria, destruct bacteria via antimicrobial agents and minimize damage to surrounding tissue, thus controlling the inflammatory response [25]. These actions suggest a more sophisticated and regulated behavior of the neutrophil cell type. The significance of NETs is demonstrated by the evasion of NETs seen by certain strains of bacteria or by the bacteria’s ability to degrade DNA, enhancing its virulence [25]. What’s more, it has been seen that mice deficient in either cathepsin G or NE (contents of the neutrophil’s primary granules and NET formation) are more susceptible to Gram-positive and Gram-negative bacterial infections, respectively [18].

The recent discovery of NETs in 2004 [60] and the knowledge gained in regards to NET formation drive the need to regulate NET extrusion upon acute confrontation with a biomaterial. Consequently, an influx of information leads to more questions than answers. What is the role of the biomaterial (composition and topography) in eliciting NET formation? Furthermore, how does the coating of the biomaterial with NETs precondition the material and alter the resulting microenvironment? What steps need to be taken to regulate this preconditioning of biomaterials?

## Neutrophils and adaptive immunity: TLR-neutrophil activation

TLR activation can lead to the production of ROS, which in turn activates neutrophils. TLRs work to detect and decode the threat (whether it is a Gram-positive or Gram-negative bacteria) and tailor the response. Neutrophils express nearly all members of the TLR family with the exception of the intracellular TLR3 and TLR7 [61]. Intracellular TLR3 and TLR7 are responsible for detection of double and single stranded viral RNA, respectively [61]. Cell surface TLRs, specifically TLR2 and TLR4, are heavily studied because they respond to Gram-positive and Gram-negative bacteria, respectively. LPS is an established TLR4 ligand and mediator of neutrophil survival by inhibiting apoptosis [61]. This effect by LPS is greater in early neutrophil activation, rather than in the late stages, lending the idea that LPS serves a protective function by reducing the neutrophil survival rate long-term in order to prevent excessive neutrophil presence which can lead to chronic inflammation, and/or to allow for excess monocyte and macrophage recruitment and infiltration [61]. Downstream factors of TLR4 impact the long-term survival of neutrophils. This is supported by a study that blocked TLR4 and consequently resolved a chronic disease situation [61]. Bacteria can activate neutrophils through intracellular TLR9 which causes the cell to perform phagocytosis, generate cytokines (specifically IL-8, TNF-α, and ROS), and even reduce neutrophil apoptosis. Interestingly, a specific fungus uses only TLR4, not TLR2, to access neutrophils [61]. Blocking of TLR4 is associated with down-regulation of the release of IL-8 and IL-10 and with anti-apoptotic behavior, which allows the fungus to persist as it sees fit. TLR4, however, does remain functional, even at very low levels of surface expression. TLR agonists have been suggested to prime the neutrophils into a persistent inflammatory state [61].

Yet another function of TLRs is their ability to detect DAMPs. DAMPs are produced by surrounding immune cells in response to an injury. These factors have been implicated in many infections, including sepsis [61]. There are DAMPs known to activate TLR2 and TLR4 which result in neutrophil activation and the continuation of the cycle of inflammation [61]. Ultimately, the regulation of neutrophil activation and removal is crucial for pathogenic clearance *in vivo*. If however, TLR activation is affected or blocked/mitigated by pathogenic presence or DAMPs, it can result in an overwhelming presence of the pathogen which can lead to a severe inflammatory response due to the constant inhibition of neutrophil recruitment, activation, and their apoptotic behavior. Moreover, excessive inflammation due to neutrophil presence can cause increased levels of secreted ROS and proteinases, which causes local tissue damage as well as induces the release of even more DAMPs [61]. This creation of an ‘autocrine loop’ can lead to the development of an autoimmune disease; therefore the activation of TLRs should be viewed and researched as a possible therapeutic intervention, especially when considering that an implanted biomaterial may become the trigger [61].

## Neutrophils and tumor formation

Both neutrophils and macrophages have been implicated in the progression of a variety of solid tumor formations [6, 34]. Melanomas, specifically, are infiltrated by NK cells, macrophages and neutrophils, comprising 80% of the present cells [34]. Tumor-associated macrophages (TAMs) and CXC-chemokines secreted by tumor cells recruit tumor-associated neutrophils (TANs) to the tumor site, and the presence of neutrophils is positively associated with the progression of tumors [34]. Inversely, the depletion of neutrophils from a tumor site has been shown to inhibit tumor growth [6]. TAMs and TANs are considered potent stimulators of angiogenesis. Neutrophil-derived vascular endothelial growth factor (VEGF) is associated with this angiogenic activity. More specifically, it has been seen that the expression of MMP-9 induced VEGF production in the TANs of tumor cells [6]. Most importantly, a more recent study has shown that one of the major, exceptionally potent, and critical tumor angiogenesis-inducing factors, MMP-9, is derived from neutrophils and not the previously thought M2 macrophages [43]. The TANs secrete a 40–50-fold increase of TIMP-free MMP-9 while invoking higher levels of *in vivo* angiogenesis relative to macrophages [43]. The role of neutrophils in tumor progression is yet another example demonstrating the plasticity of neutrophils in response to received environmental signals.

**Figure 4.**
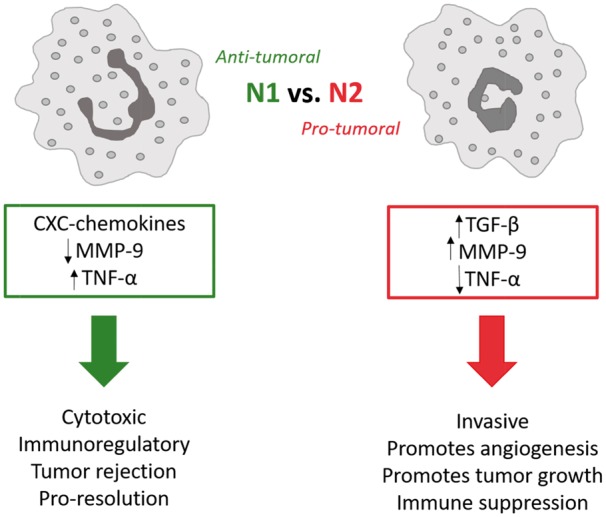
Schematic relays the distinct differences between N1 (anti-tumoral) and N2 (pro-tumoral) neutrophils.

Thus, neutrophils can play two opposite roles in tumor biology. As leukocytes, they can behave in a protective manner by killing tumor cells; it has been seen that tumor reduction can be as great as 49% upon exposure to an influx of neutrophils [34]. In contrast, neutrophils are also enormously involved in tumor formation and progression by releasing growth signals, MMPs, and mediators of angiogenesis [34]. These vast differences, highly regulated by the microenvironment, lead to the polarization of neutrophils to two distinct phenotypes: N1 (anti-tumoral) vs. N2 (pro-tumoral) neutrophils, analogous to the M1 and M2 macrophage phenotypes [2, 62]. And as such, these distinct phenotypes can influence the microenvironment to either facilitate tissue integration of the biomaterial or promote chronic inflammation (Fig 4).

### N1 vs. N2 phenotype in activated neutrophils

N1 neutrophils are anti-tumoral and are characterized by hypersegmented nuclei, increased expression of TNF-α, a lack of or reduction in production of VEGF and MMP-9, which are key to angiogenesis, and therefore related to tumor progression [34]. The immunosuppressive cytokine, transforming growth factor-β1 (TGF-β), plays a distinguishing role in determining the neutrophil’s polarization fate; the sustained presence of TGF-β results in N2 polarization and, consequently, a blockage of TGF-β results in N1 polarization with immune-activating cytokines and chemokines which have enhanced killing tendencies [34]. N1 neutrophils tend to be cytotoxic (tumor rejection) and play a role in immune memory [34]. In contrast, the N2 neutrophils are pro-tumoral, exhibiting tendencies to be invasive, promote angiogenesis and tumor growth, and suppress the immune system [3, 19, 34, 62]. N2 neutrophils produce more MMP-9 (Table 1), specifically TIMP-free MMP-9, which is an extremely potent stimulator of angiogenesis, critical to matrix remodeling for tissue/biomaterial integration [63]. TIMP-free MMP-9 release can also promote tumor growth and metastasis [34]. Additionally, in many tumors, the cancer cells are responsible for none of the matrix-degrading proteins (MMPs), leaving that up to the inflammatory cells like the N2 neutrophil [34]. The N1 vs. N2 polarization (Fig. 3) of the neutrophil is critical to its role as central player of inflammation and resolution and identifies an importance in controlling the N1/N2 ratio; the N1/N2 ratio may in fact be just as critical as the M1–M2 ratio in regulating tissue integration. Thus, utmost attention in regulating the acute confrontation between the neutrophil and biomaterial will serve to harness overall inflammation, regulate neutrophil phenotype, and promote biomaterial integration.

## Neutrophils and lipoxins

Lipoxins are lipid mediators and play an important role as ‘braking signals’ to resolve inflammation [64]. Activated platelet adhesion to neutrophils is an abundant source of lipoxins. Lipoxins are produced in response to stimuli and act in a local manner before becoming metabolically inactive rather quickly. Leukocytes express recognition sites (G-PCRs) for lipoxins, one of the most important receptors found on human neutrophils [64].

Lipoxins can act as anti-inflammatory molecules by inhibiting neutrophil recruitment and activation and can even stimulate monocyte chemotaxis, which does not cause monocyte degranulation or ROS release [64]. Additionally, nearing the end of acute inflammation, neutrophils can alter the synthesis of LTB4 to lipoxin A4 (LXA4) (a pro-resolving lipid mediator) which inhibits neutrophil recruitment because it interacts with the G-PCR LXA4 receptor (LXA4R). LXA4 is also associated with reduced inflammation and ultimately the resolution of the inflammatory response and regenerative pathway by influencing macrophage phenotypic shift from M1 to M2, resulting in increased release of TGF-β1 from the macrophages and a decrease in release of monocyte chemoattractants [64]. This demonstrates the dynamic plasticity of the neutrophil as it adapts its phenotypic pathway, switching from synthesis of LTB4 for NE production in earlier stages to the production of LXA4 for resolving purposes in later stages. In this way, neutrophils can contribute to the formation of resolvins and defensins through the activation of M2 macrophages [6]. Lipoxins, resolvins, and defensins can also increase the uptake of apoptotic (or post-NETosis) neutrophils via macrophages [6, 18, 65]. Therefore, it is evident that the presence of LXA4 may signal the regenerative pathway and the appropriate cell recruitment, like the M2 macrophages. What can be done to promote LXA4 production in the microenvironment? One group has researched aspirin as a potential solution to increasing the presence of LXA4 [66].

When the neutrophils give off eat-me signals, there is a triggering of a specific phenotype in the engulfing macrophage, polarizing the resulting macrophage to an M2 repair-like phenotype which proceeds along a regenerative pathway, negatively regulating inflammation [6]. These functions are especially critical in regards to biomaterials as the resulting shift to M2 repair-like phenotype of macrophages promotes resolution and matrix reprogramming, culminating in enhanced implant integration. This shift is crucial to host-biomaterial integration as the switch to a more regenerative-like environment should allow for incorporation of the biomaterial, and it is becoming clearer that the synergy of multiple cell types and mediators is the key to resolution and that ‘nearly every necessary process is aided by or requires the neutrophil’.

## Neutrophils and macrophages: the crucial pairing

As briefly mentioned earlier, neutrophils are extremely important for activating and recruiting macrophages to the site of inflammation, and the cycle continues with the macrophage recruitment of neutrophils. When activated, neutrophils release macrophage inflammatory protein-1α (MIP-1α), MIP-1β and IFN-γ [18]. Macrophages can engulf both non-apoptotic and apoptotic neutrophils as well as MPO (specifically the macrophage mannose receptors) from the neutrophils [11]. Although the uptake of apoptotic neutrophils results in an M2 repair-like phenotype of macrophages, the uptake of MPO results in the release of even more ROS and pro-inflammatory cytokines, resembling an M1-like macrophage phenotype [18, 65]. This ingestion by the macrophages is critical to the control of inflammation and ultimately, resolution [11]. Additionally, macrophage release of TNF-α, G-CSF and granulocyte macrophage-CSF increases production and recruitment of neutrophils, as well as inhibits apoptosis of neutrophils, thereby extending their life span at the wound site from 6–12 to 24–72 h, enabling further synergy between the two cell types [4, 11, 18].

As previously discussed, the interaction of neutrophils with lipoxins can leverage recruited macrophages to behave in an M2 repair-like manner producing resolving mediators and cleaning up the area by engulfing and ridding the area of apoptotic neutrophils, which is crucial and necessary for regeneration to take place [67]. This is often associated with M2 macrophage release of resolvins and defensins but also with the release of MMP-9 which is directly related to increased angiogenesis and neovasculature formation. This phenomenon is highly indicative/symbolic of matrix reprogramming and ultimately tissue remodeling, which are both hallmarks of resolution and host integration of the biomaterial. Not only is the role of the macrophage as an M2 important, but the pure removal of the neutrophils from the inflammatory site is critical for resolution of the inflammatory response as the neutrophil recruits and stimulates a multitude of inflammatory and immune cells [6]. Simply removing the cell type from the conflicted area by phagocytosis will result in a shift in the ratio of neutrophils to macrophages during which macrophages can become the more predominant cell type and recruit new cell types such as fibroblasts to begin laying down new matrix [6]. Although it is still important to remember the maintenance of the M2/M1 ratio in macrophage presence as the M1 is associated with chronic inflammation and the M2 with regeneration, it is a perpetual balancing act of the two, and from this evidence, is heavily influenced by the neutrophil.

Excessive macrophage presence and the M1 phenotype is implicated in the development of a chronic inflammatory response and eventually, a fibrotic response when related to fibrous (scar) tissue formation or fibrotic encapsulation of a biomaterial. Although M1 macrophages release pro-inflammatory factors, the M2 macrophages release MMP-9 and anti-inflammatory mediators such as IL-10 leading to matrix reprogramming [67]. M2 macrophages are present in resolved tissue regeneration and a phenotypic switch from M1 to M2 is a possible strategy for mitigating the effects of inflammation [67]. Furthermore, it has been seen that biomaterials which are synthetic and slowly degrading become fibrotically encapsulated in a matter of weeks through the development of frustrated macrophages and formation of foreign body giant cells. However, when porous synthetic materials have been used, the material elicited an M2 response from macrophages and thus shifted the M2/M1 ratio of macrophages present, resulting in healing and resolution with little to no fibrosis [67]. Moreover, analogs produced from naturally derived materials such as ECM analogs or decellularized tissue constructs have been known to cause a noticeable switch of an M1/M2 macrophage phenotype, presence of infiltrated macrophages, and neovascularization most likely due to the topography, pore size and interconnectivity of the pores, and the material’s inherent surface functionality [67, 68]. This underlines the importance of the biomaterial composition, fabrication and topography as they work in tandem with neutrophils in regards to inducing a pro-resolution macrophage phenotype. Previous work with macrophages and the M1 vs. M2 phenotype have outlined important aspects and phenotypic behaviors of immune system cells, broadening the research community’s curiosity with regard to other cell types. Although the recognition and importance of the macrophage’s role has been well-established, it has become clear that if the macrophage’s role is impacted by preceding and concurrent cells and the resulting microenvironment, then the research must back up and begin with the neutrophil. In doing so, and with the abundant knowledge on macrophages already available, investigators can begin to unravel the synergistic, multifaceted roles of the innate immune system cells in response to biomaterial implantation.

## Neutrophils and fibrosis

Fibrosis is characterized by four steps: initiation of the host response, always in the case of injury, activation of effector cells, elaboration of the ECM and deposition of and lack of removal of ECM that progresses to fibrosis and organ failure [69]. Fibrosis is an active and dynamic process, plastic in nature, changing based on the effector cells and chemical signaling. Although typically permanent, it has been seen that some fibrotic response is reversible. If considered a dynamic, active process, it can be managed to alter and change course and regress [69]. Both acute and chronic inflammation can cause fibrosis. Macrophages play a role in interstitial fibrosis via involvement in the TGF-β pathway (secreted by inflammatory and effector cells acting in both an autocrine and paracrine manner), but can also be protective through their engulfment of apoptotic cells [that promote inflammation] and fibrogenic agents as well as the release of MMPs [69].

Fibroblasts and myofibroblasts are also key cells in the fibrotic response as they are responsible, in large, for new matrix deposition. Collagen types I and III are produced by these cells where a greater ratio of I/III is associated with a greater fibrotic tissue formation. Myofibroblasts, indicated by their name, express smooth-muscle proteins and contribute to fibrotic scarring by smooth-muscle cell like contraction. Biomaterials must be designed such that this distortion of matrix is minimized. The excessive formation of collagen and tissue contraction results in reduced tissue function leading to fibrosis, indicating an appropriately timed presence and removal of myofibroblasts is an important aspect of evading fibrosis [70]. Interestingly, macrophages have been implicated as a possible source of myofibroblasts at injury sites by means of transdifferentiation [70]. What, then, is the connection between the neutrophil and immune effector cells (i.e. macrophages) and with the myofibroblasts? How can this be modified or mediated through biomaterial design?

## Biomaterial design considerations and desireable outcomes

### Priming the biomaterial

An important concept to consider for the role of the neutrophil in biomaterials integration and regeneration is the ability of the neutrophil to precondition or prime the biomaterial for the following immune cells through its release of chemical factors and NETs. As the powerful functionality of the neutrophil has been described, it is easy to imagine that the neutrophil, which is the first cell type interacting with the material, and its response, which is largely induced by the biomaterial and microenvironment, may drastically impact the subsequent series of events. We know that the neutrophil will respond in different capacities depending on the cues given to it [6, 20, 23, 25, 27, 28, 30, 32, 34, 61, 62]. Given one set of cues, the neutrophil may function primarily as an orchestrater, cleaning up the biomaterial and surrounding microenvironment and secreting regenerative factors to facilitate repair. With another set of cues, the neutrophil may become a disrupter, extruding excessive NETs decorated with damaging factors and secreting inflammatory signals. In either scenario, the biomaterial will be primed by the neutrophil for the following response in a manner not previously recognized. Taking this into consideration, it is now clear that it is vitally important to investigate the neutrophil’s critical role as the first responder, preconditioning the biomaterial, if the goal is to design the optimal biomaterials for the intended applications.

### Biomaterial design

Taking into consideration the level of importance of the neutrophil to the regulation, activation and resolution of the immune system, it is clear that this particular cell type is the key effector cell, or the ‘Cinderella’ of the immune system [18]. Therefore, the design of biomaterials should be centered on the neutrophil first with a focus on modulating the neutrophil activation and response to elicit the subsequent appropriate response of the remaining effector cells including monocytes, macrophages, etc. [46].

As discussed in parts throughout this review, the immune system response to a biomaterial implantation can be an acute inflammatory response and resolution or chronic inflammation. Research has shown that synthetic, non-porous biomaterials with low surface area to volume ratio more often result in sustained inflammation, characterized by excessive neutrophil and macrophage presence, excessive NET extrusion, the presence of foreign body giant cells, and fibrotic encapsulation. Specifically, *in vitro* and *in vivo* research has shown that certain surface chemistries impact monocyte and macrophage adhesion and fusion; hydrophilic, non-ionic and anionic surfaces significantly down-regulate adhesion macrophage fusion to form foreign body giant cells [1]. Moreover, leukocyte adherence, adherent leukocyte cytokine production, and non-adherent leukocyte exudate (mouse cage implant system) have been evaluated as a function of surface chemistry with regard to biomaterial implant to determine whether specific surface chemistries influenced a pro- or anti- inflammatory microenvironment [71]. Analysis of four surface chemistries (hydrophilic, hydrophobic, anionic and cationic) revealed that leukocytes adhered to hydrophilic surfaces exhibited significantly decreased production of pro-inflammatory cytokines (IL-6, IL-8) relative to a base surface and that the same hydrophilic surfaces had significantly decreased levels of leukocyte adhesion and foreign body giant cell fusion [71]. Conversely, hydrophobic and cationic surfaces had significantly increased levels of leukocyte adhesion as well as macrophage fusion to form foreign body giant cells. Hydrophilic surfaces, specifically, down-regulated the production of pro-inflammatory cytokines by both adherent and exudate cells [71]. These results inform the researcher that surface chemistries need to be considered because the functional profiles of cells can be influenced accordingly. Therefore, hydrophilic and non-ionic biomaterials have the potential to limit leukocyte adhesion, limit macrophage fusion to form foreign body giant cells, and promote regenerative inflammation.

Many aspects of design have been implicated in influencing the modulation of neutrophil phenotype from pro-inflammatory to pro-resolution, including fabrication (i.e. porous and fibrous materials or solid materials) and composition of these materials. It seems that it would only be logical to consider using an ECM analog composed of a naturally derived polymer in order to maintain some of their material’s natural functionality, whether it be proteins or platelet fragments, growth factors, etc. The microstructure of the material is also tantamount to the neutrophil activation and can be responsible for neutrophil and macrophage interaction and infiltration as well as the formation of new blood vessels or angiogenesis. The ECM is composed of nanofibrous networks with resulting micro pores for cell infiltration and migration. And it has been seen, as discussed earlier, that there is a positive inflammatory response that eventually leads to resolution of inflammation and regeneration with no fibrosis response when porous, fibrous and naturally derived materials were used [67]. Therefore, a focus of the material should be largely its fabrication method.

If the objective of the structure is to create an ECM analog, it should be considered necessary to mimic the nanofibrous nature of ECM by use of fabrication methods such as electrospinning. Electrospinning allows tailoring the fabrication parameters to meet the needs of the specific tissue type, whether it be an aligned or random fibrous network native to that tissue type, keeping in mind that although some tissue types are highly aligned and oriented, this too can elicit an inflammatory response from the neutrophils. The high surface area to volume ratio of implants such as fabrics or porous materials have higher ratios of macrophages to foreign body giant cells at implant sites rather than smooth surface implants which have significant fibrosis at the implant site [22]. This observation is directly related to the need for a porous, highly interconnected electrospun construct as it is expected to allow for macrophage infiltration and not frustrated phagocytosis.

### Neutrophil-template interaction

**Figure 5.**
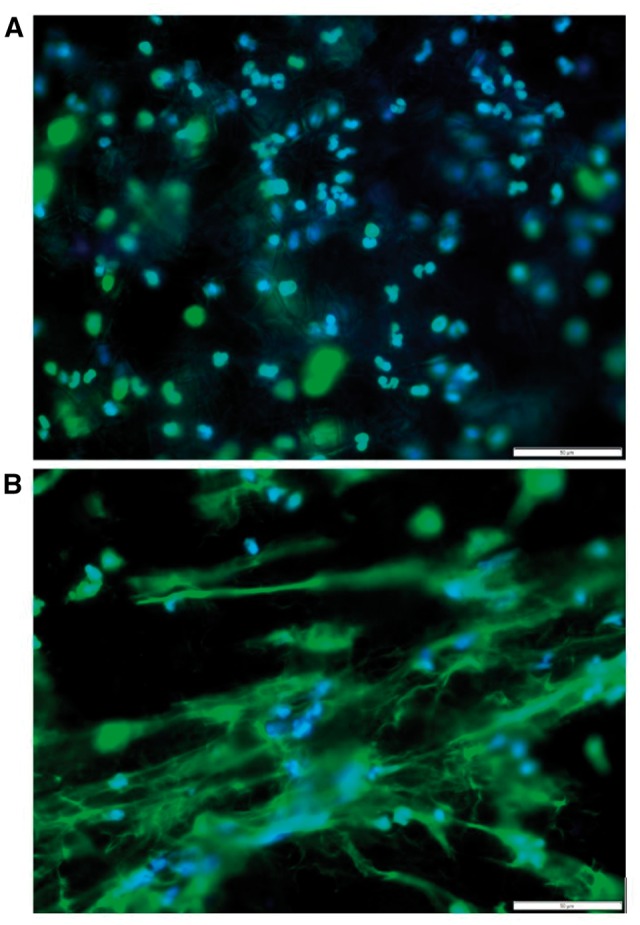
Representative fluorescent images of freshly isolated neutrophils seeded onto polydioxanone electrospun templates at 3 hrs. Top panel a is a large fiber diameter (1.9 ± 1 µm) template while bottom panel B shows a small fiber diameter (0.3 ± 0.1 µm) template eliciting a greater amount of NET extrusion. The stains utilized are DAPI (blue) for nuclei and SYTOX green (green) for extracellular chromatin, or NETs. For both images, magnification is 40× and scale bar is 50 µm.

A preliminary study was conducted with the goal to begin to answer the questions regarding the role of the biomaterials in eliciting NET extrusion and preconditioning the biomaterial. Freshly isolated human peripheral blood neutrophils were seeded onto two topographically different materials of the same composition (Polydioxanone (Sigma-Aldrich, St Louis, MO)). Following a 3-hr neutrophil-biomaterial interaction, the templates were fixed in formalin and stained with both 4′,6-Diamidino-2-Phenylindole, Dihydrochloride (DAPI) (for nuclei) and SYTOX green (for extracellular DNA) fluorescent stains to detect the formation of NETs in response to the materials. Figure 5 communicates the results of the brief study and analysis and visually highlights the stark differences in neutrophil behavior when exposed to two significantly topographically different Polydioxanone ECM analogs achieved via electrospinning.

The results were such that the electrospun material with a large fiber diameter (1.9 ± 1 µm) attracted neutrophils which remained intact with minimal NET extrusion. In contrast, the electrospun material with a small fiber diameter (0.3 ± 0.1 µm) elicited a massive NET formation in response. This initial finding suggests that NET extrusion is indeed regulated by biomaterial template topography. Although an important finding, it obviously creates more questions than answers, and yet again, encourages the need to examine and regulate the acute confrontation of neutrophils and biomaterials. Such inspection shall grant understanding of neutrophil pre-conditioning of biomaterials.

Biomaterial size and structure, and even pathogen size and structure have been found to elicit varying degrees of neutrophil response. Jhunjhunwala et al. found that present neutrophil number was greater in response to cross-linked alginate implants when compared to the response when alginate was injected as solution, indicating that the structure of the implant alone, can induce greater cell recruitment [47]. In another interesting finding, Branzk et al. reported that NET release was influenced by fungal and bacterial morphology, not just presence alone; when the pathogen was small enough to be phagocytosed, no NETosis was induced; however when the same pathogen was present in larger aggregates (i.e. hyphae), NETosis was induced [54]. Together these findings continue to lead to questioning the role of the 3D template architecture in regulating neutrophil behavior, NETosis, and priming of the biomaterial.

The material composition should also attempt to closely mimic the materials native to the tissue type, whether it be collagen types I–III or fibrinogen, platelet rich plasma (PRP) etc. However, much consideration would need to be made when using a naturally derived polymer as they tend to degrade rapidly both *in vitro* and *in vivo* and therefore may not be appropriate choices on their own. This would lead to the consideration of the combination, or hybrid, of natural and synthetic polymers, choosing carefully the synthetic polymer for both its degradation rate and mechanical properties, such as modulus of elasticity, tensile and compressive strengths, and behavior under the specific mechanical stresses of the region for which the construct is being developed. Many research groups consider adding factors to the polymeric solutions for contribution to the composition of the ECM analog in order to better induce cell infiltration, migration, and ultimately MMP destruction and reprogramming; growth factors, PRP, specific proteins and such could be added, but in the interest of activating and recruiting neutrophils, a strong suggestion could be the incorporation of aspirin as it has been known to induce a LXA4 presence, which modulates neutrophil activation and can aid in resolution of the inflammatory response [66]. As such, the timing of the release of the aspirin would need to be closely monitored as premature release could inhibit the necessary steps of inflammation and neutrophil and macrophage recruitment, but too delayed of a release may be ineffective.

There are many unknowns regarding the capabilities of neutrophils and biomaterial activation of the cell type. Although many tissue engineering aims have focused on tissue regeneration, it has predominantly been focused on either avoiding or destroying activated neutrophils and/or eliciting a specific response from macrophages. It has not been until recent years that the neutrophil and its biology was even discovered, let alone, considered an emerging research topic in the area of tissue engineering and biomaterials. Therefore, researchers and scientists in the field are missing vital information about the activation of neutrophils, how to properly induce the appropriate formation of NETs, and harness the macrophage recruitment to the site to engulf said apoptotic NET producing neutrophils in order to generate a more M2-like macrophage phenotype typically suited for resolution of inflammation and tissue regeneration. The limitations and concerns encompass excessive presence or activation of neutrophils leading to a multitude of problems, such as chronic inflammation and tumor formation, exactly the opposite of the desired response. Inappropriate activation and continuous recruitment of neutrophils can result in tumor formation and progression, chronic inflammation, sepsis, or full rejection/failure of the implanted material. There has to be a switch that allows the neutrophils to act as pro-inflammatory agents and then resolution-focused agents through NETosis and macrophage recruitment. As essential as it is to harness the capabilities of the neutrophil, it is ever more essential to be able to terminate it.

Neutrophil research is most likely heavily hindered due to the short life of the cell type and lack of established cell lines but also due to the ‘lack of knowledge and acceptance of the neutrophil as the true maestro of the immune system’, evident by research continuing to focus on macrophage differentiation, activation and modulation [4, 22]. Although important, macrophages are not the starting point. The biology of neutrophils is only of recent concern with researchers, and as such there is an insufficient number of studies utilizing the neutrophil and harnessing the body as a bioreactor to promote *in situ* regeneration. There have been reviews and introductions to the many different activators of neutrophils, explanations of the process of NETosis, but little to no exploration of this vital cell type’s utilization in a treatment or therapeutic nature. As discussed throughout this review and further highlighted in Figure 5, neutrophil behavior needs to be incorporated in analysis of biomaterials research by including the cell interaction with templates, NETosis evaluation, and possibly co-culture systems with monocytes and macrophages, or multi-step culturing where the template interacts with neutrophils, and subsequently macrophages as this is the reality of the phases of the innate immune system's wound healing response.

## Shifting the focus to the neutrophil

Taken together, it is clearly evident that it is not just desirable, but necessary, to activate and induce a specific and appropriate response from neutrophils upon implantation of a biomaterial. The neutrophil is heavily involved in both the initiation and amplification of the inflammatory response; therefore neutrophil behavior and NETosis regulation is critical to template success. Neutrophils and NETs can either fight or cause disease/destruction; it is the researcher’s role to understand this neutrophil-biomaterial interaction to tailor the design to utilize the neutrophil’s regenerative capabilities [22]. This review has outlined the potential of the neutrophil to balance the promotion of regeneration and demotion of inflammation through regulating phenotype, cell recruitment, NETosis, and chemokine/cytokine production. This brief introduction of neutrophils’ many important roles in inflammation and resolution does not even begin to scratch the surface of the orchestrated and well rhythmed series of events that occur along the regenerative pathway, but it does lend to persuade the reader of the importance of harnessing the capabilities of this central player when designing, fabricating, and evaluating host-biomaterial integration and regenerative possibilities.

## Funding

This work was supported by the Memphis Research Consortium.

*Conflict of interest statement*. None declared.
